# The Cost of Activity during the Rest Phase: Animal Models and Theoretical Perspectives

**DOI:** 10.3389/fendo.2018.00072

**Published:** 2018-03-07

**Authors:** Antonio A. Nunez, Lily Yan, Laura Smale

**Affiliations:** ^1^Department of Psychology and Neuroscience Program, Michigan State University, East Lansing, MI, United States; ^2^Department of Integrative Biology, Michigan State University, East Lansing, MI, United States

**Keywords:** shift work, circadian rhythms, temporal niche, eveningness, grass rats

## Abstract

For humans, activity during the night is correlated with multiple pathologies that may reflect a lack of harmony among components of the circadian system; however, it remains difficult to identify causal links between nocturnal activity and different pathologies based on the data available from epidemiological studies. Animal models that use forced activity or timed sleep deprivation provide evidence of circadian disruptions that may be at the core of the health risks faced by human night and shift workers. One valuable insight from that work is the importance of changes in the distribution of food intake as a cause of metabolic imbalances associated with activity during the natural rest phase. Limitations of those models stem from the use of only nocturnal laboratory rodents and the fact that they do not replicate situations in which humans engage in work with high cognitive demands or engage voluntarily in nocturnal activity (i.e., human eveningness). Temporal niche switches by rodents have been observed in the wild and interpreted as adaptive responses to energetic challenges, but possible negative outcomes, similar to those associated with human eveningness, have not been systematically studied. Species in which a proportion of animals shows a switch from a day-active to a night-active (e.g., grass rats) when given access to running wheels provide a unique opportunity to model human eveningness in a diurnal rodent. In particular, the mosaic of phases of brain oscillators in night-active grass rats may provide clues about the circadian challenges faced by humans who show voluntary nocturnal wakefulness.

## Human Night Work

Our contemporary global society has created demands that require many of us to be active during the natural rest phase of our species, the night. This is exemplified by increasing number of individuals who work nights and thus are awake and engaged with the environment at least for part of the time normally dominated by human sleep. There is a rich, albeit mostly correlational, literature linking night and shift work to a multitude of pathologies including higher risk of cancer (1), metabolic syndrome (2), hypertension (3), cognitive deficits (4), and female infertility (5), among other health and behavioral problems. Many of these negative outcomes may stem from a lack of harmony among different components of the circadian system (6).

The circadian system consists of a principal oscillator located in the hypothalamic suprachiasmatic nucleus (SCN) (7, 8) that entrains to the light–dark cycle *via* direct retinal projections from melanopsin-containing retinal ganglion cells (9). Outputs of the SCN serve to synchronize a multitude of extra-SCN oscillators in the brain and in the peripheral organs (10). Night or shift work is associated with exposure to environmental influences that challenge the temporal regulation of many behavioral and physiological functions. When undisturbed, the resulting daily rhythms are kept at optimal phase relations among themselves and synchronized (entrained) to the 24-h day–night cycle by the circadian system. Some of the challenges faced by the circadian system of night workers include exposure to nocturnal light, activity during the natural sleep period, and food consumption during the rest phase of the cycle. It is very likely that many of the health and behavioral problems of night or shift workers stem from circadian disruptions resulting from external (light) and internal (metabolic signals; release of neurotransmitters) stimuli that interfere with, or override, circadian signals emanating from the SCN (6, 11). However, it is difficult to draw conclusions about causality from epidemiological studies with humans. A number of animal models have been developed to circumvent those limitations.

## Animal Models of Night Work: Insights and Challenges

Animal models of shift work have used almost exclusively laboratory rodents (see Ref. (12) for a review) that are forced to be active during their rest phase by placing them in rotating wheels for several hours every day (13–15) or alternatively, by keeping them awake during the rest phase using gentle stimulation whenever the animal gives signs of falling sleep (16). Forced activity for 8 h/day during the normal rest phase of laboratory rats, sustained for five consecutive days per week, results in increased abdominal fat accumulation and the display of several indicators of metabolic syndrome, including impaired glucose tolerance (13). These animals also shift their food intake to the light period (13, 14) and show reduced general activity as well as a reduced activity-rhythm amplitude on days off from the forced activity regime (17). Interestingly, providing food only during the normal active (dark) phase prevents many of the effects of forced activity during the light phase (15), and at least in studies using rats, restricting feeding to the light phase in otherwise undisturbed animals mimics the effects of the forced activity manipulation (15). A different study that used a forced activity paradigm similar to that of experiments reporting increases in body weight and adiposity, surprisingly found a reduced body weight in the shift-working rats (18). Differences in housing conditions or stress level of the animals could be responsible for the different outcomes, but of note is the observation that in these shift-work animals that lost weight, the amount and distribution of activity on days off did not differ from those of control animals not exposed to the forced activity regime. Thus, both changes in energy expenditure and the emergence of day-time feeding appear to contribute to the metabolic effects of forced activity during the normal rest phase of nocturnal laboratory rats.

There is ample evidence that sleep deprivation *per se* can negatively affect metabolism and energy balance (19, 20). Experiments in which chronic timed sleep restriction, with opportunity for sleep recovery within the 24-h period, are most relevant as animal models of shift work. Several studies using mice have reported metabolic deficits including abnormal glucose and lipid metabolism when the animals are deprived of sleep during the first 6 h of the night for two blocks of 5 days separated by 2 days of *ad lib* sleep (16, 21). Restricting feeding to the night prevented these metabolic effects (16); however, different from what was reported for rats, restricting feeding to the light phase without sleep restriction did not result in metabolic anomalies (16). Also different from most of the forced activity work with rats, sleep restriction did not affect body weight in mice (16).

Although studies using forced timed activity or timed sleep restriction provide causal links between the human experience of night shift work and circadian, metabolic, and energy disruptions reported for these workers, they have some clear limitations. First, the use of nocturnal laboratory rodents poses questions about how generalizable the findings are to diurnal species such as ours. Also, even within nocturnal rodents, the limited data hint at possible differences between laboratory rats and mice (12), some of which likely stem from differences in body size and how that affects responses to metabolic challenges. Using a wider range of species, including day-active (DA) ones, would add significantly to the value of animal models of human shift work that use forced activity or timed sleep restriction.

## Human Eveningness

Although humans are clearly diurnal, many of us become active during our normal rest phase, the night. This change in phase preference is not limited to those engaged in shift or night work. For example, many young adults shift their activity phase and display what is known as “eveningness,” which involves being active during a large proportion of the night. There is compelling evidence that in humans, voluntary shifts to a nocturnal activity profile result in substantial negative outcomes, including eating disorders (22), diabetes, and metabolic syndrome (23). Further, eating at the inappropriate phase has been linked to obesity in humans and animals (24, 25). These problems have clear negative impacts on the human capital of society. The animal models that simulate human shift work using forced activity or timed sleep deprivation (see above) are not ideal to study the consequences of the apparent voluntary temporal niche switch of human eveningness. For example, the expression of Fos protein in the brain of grass rats (*Arvicanthis niloticus*) is remarkably different if the animals are forcibly kept awake at night compared to when they show unconstrained night wakefulness (26, 27) (more about this animal model below). Interestingly, shifts in the balance between day/night activity, like those seen when eveningness emerges in teenagers, have been reported for other mammalian species, both in the field and in the laboratory (28–30) and thus represent potential models for understanding the causal links between human voluntary nocturnal activity and the negative outcomes associated with it.

## Mammalian Temporal Niche Switches

Mice, which are strictly nocturnal in standard laboratory conditions, can switch to diurnality when observed under more natural conditions for extended periods of time (31). An influential hypothesis to account for these switches by mice postulates that they occur in response to energy challenges (32). Specifically, this perspective suggests that situations in which animals experience negative energy balance favor the display of a diurnal phenotype (33). Laboratory work testing this hypothesis has used a “work for food” paradigm in which mice get food only if they run in a wheel, thus emulating the foraging demands of the wild (34). The workload to obtain a particular amount of food is manipulated to resemble environments with different densities of resources. Under those conditions, increasing the workload induces a phase advance of the activity (or work) rhythm, such that normally nocturnal mice show predominantly diurnal activity (32). Reduced ambient temperature, while kept with *ad lib* food availability, also induces a shift to diurnal activity, and enhanced workload and low ambient temperature challenges have additive effects with respect to this temporal niche switching in mice (35). The change in the phase preference for the display of activity when mice experience a negative energy balance is not accompanied by a shift in the phase of the SCN oscillator, but peripheral oscillators in the liver and adrenal gland show a phase that more closely resembles that of diurnal mammals (35).

The thermoenergetic hypothesis advanced by Hut and coworkers (30, 32) suggests that diurnality emerges in rodents to reduce energy needs, since days are warmer than nights. While this hypothesis promotes the adaptive value (32) of the temporal niche switch (31), there may also be costs, as activity during the natural rest phase of the mice was accompanied by changes in synchrony of internal rhythms. Specifically, peripheral oscillators shifted their phase angles with respect to the SCN and likely with respect to the melatonin rhythm, which remains nocturnal in other models of temporal niche switching (29, 36).

## Temporal Niche Switches in Diurnal Species: The Grass Rat as a Model

There are examples of species that are diurnal in the field, but that switch to a nocturnal activity profile in the laboratory (37). Based on the thermoenergetic hypothesis, these observations suggests that these animals may be exposed to energetic challenges in the wild that favor diurnality and that do not exist in the laboratory. More interesting from the perspective of developing a model for human eveningness are species, e.g., Nile grass rats (28) and *Octodon degus* (29), sometimes referred to as dual-phasing animals (29), which are diurnal in the field, and also under standard laboratory conditions, but can show either diurnal or nocturnal phenotype when given access to running wheels (28).

For over 20 years, our group has been developing the grass rat as a diurnal mammalian animal model to study the circadian system (38). To go with their diurnal life style, grass rats feature an abundance of retinal cones (39) and an optic tectum that, relative to body size, is four times the volume of that of laboratory rats (40). Although the phase of the SCN oscillator with respect to the light–dark cycle is similar to that of nocturnal rodents (41), brain and peripheral extra-SCN oscillators, monitored using the pattern of expression of clock gene products, are 180° out of phase in reference to those of nocturnal rodents (41, 42). Interestingly, and pertinent to the discussion of human eveningness, with access to running wheels, some grass rats switch to a predominantly nocturnal display of wheel-running activity. There is evidence of a “compromise” in night-active (NA) grass rats between diurnal tendencies and the display of activity during the normal rest phase of the species. Thus, NA grass rats keep several features of their diurnal profile including the display of frequent sleep episodes and low body temperatures late in the night (28, 43) like those seen in DA animals. This interval of sleep is followed by a pre-dawn peak of activity common to both chronotypes (28). Moreover, even though the NA animals sleep more during the day to recover from the sleep debt created by their nocturnal activity, their day-time sleep is fragmented with relatively short sleep-bout lengths (43).

**Figure 1:**
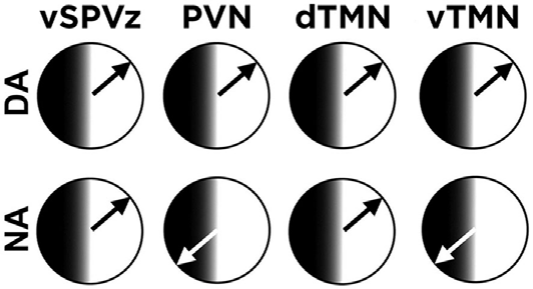
Mosaic of phases of the PERIOD 1 rhythm in hypothalamic extra-suprachiasmatic nucleus (SCN) regions: The phase of the rhythm is similar between day- and night-active (DA and NA, respectively) grass rats for the ventral subparaventricular zone (vZPVz) and the dorsal tuberomammillary nucleus (dTMN). In contrast and similar to most extra-SCN oscillators outside the hypothalamus, the rhythm is 180° out of phase between DA and NA grass rats in the paraventricular nucleus (PVN) and in the ventral tuberomammillary nucleus (vTMN). See text for references.

The retention of some diurnal tendencies in NA grass rats may be due to the diverse responses of extra-SCN brain oscillators to the switch to nocturnal activity. Perhaps not surprisingly, the adoption of a NA profile does not affect the phase of clock gene [PERIOD 1 and 2 (PER1/2)] rhythms in the SCN (41) or the nocturnal production of melatonin (36). However, most extra-SCN brain regions that express rhythms in PER 1/2 display a complete reversal of the time of peak expression when grass rats become NA, thus making the circadian profile of NA grass rats similar to that of nocturnal rodents (41). But, the reversal is not universal, again revealing features that are retained by NA grass rats from their antecedent diurnal profile. Outside the hypothalamus, the central amygdala shows a very similar pattern in NA and DA grass rats that contrasts with what is seen in the rest of the extrahypothalamic brain (41). In the extra-SCN hypothalamus (Figure 1), the paraventricular nucleus (PVN) shows a phase reversal in NA grass rats (36), but the ventral subparaventricular zone remains fixed and similar in phase to that of DA animals (41). Most relevant for understanding the sleep fragmentation of NA grass rats during the day is the response of the hypothalamic histaminergic nuclei [i.e., the dorsal and ventral tuberomammillary nuclei (dTMN and vTMN), respectively] to the switch to nocturnality in these animals. Like the PVN, the oscillator of the vTMN of NA grass rats shows a phase reversal, but in sharp contrast that of the dTMN, it retains the phase typical of DA animals (44). These results suggest that the fragmented recovery sleep of NA grass rats results from a mismatch between rhythms in components of the histaminergic arousal system of the tuberomammillary nuclei (45) and the rest/activity cycle. The work with NA and DA grass rats suggests that although temporal phenotypes are flexible and may change in service of energy homeostasis, those changes are not likely to be complete. The compromises between diurnal and nocturnal features of NA grass rats most likely reflect an internal circadian desynchrony that may be an additional cost paid by humans who voluntarily adopt a nocturnal profile. It would be instructive to determine if temporal niche switches in the wild (31) are also associated with similar circadian costs to accompany risks due to exposure to different competitors and/or predators for whom they lack preparation (32).

## Future Considerations

Available models using constrained (forced activity and timed sleep deprivation) or unconstrained (wheel running availability) activity during the natural rest phase of a species do not incorporate the effect of the type of engagement with the environment that goes on during the new active phase. In particular, these models do not replicate instances of human nocturnal activity with significant attentional and cognitive demands, e.g., nurses in hospitals or technicians working at nuclear plants. Experiments in which nocturnal laboratory rats are trained and tested during the day on tasks that demand enhanced attentional performance provide evidence of clear circadian effects that include a shift to a predominantly diurnal chronotype with salient anticipatory activity that persists for days after the training is discontinued (46, 47). Activities with low cognitive demands, such as spatial learning or training using operant tasks with low attentional requirements, do not substantially affect circadian activity; neither do daily handling or restriction of water availability to the light phase. (46, 47). The circadian effects of high cognitive-demand tasks are likely mediated by cholinergic inputs to the SCN (11), which may affect the nucleus in ways different from other forms of temporal niche switches. Determining the effects on metabolism and energy balance of different activities during the rest phase, with varied cognitive demands, would add significantly to the value of animal models of human night work or eveningness. Also important to consider when assessing the cost of constrained or unconstrained switches in temporal niche is the influence of circadian phase on cognitive competence. Thus, just like cognitive effort can affect the temporal distribution of activity, time of day can affect the acquisition (46) or retention of learned tasks (46, 48). How cognitive functions may be affected by changes in the preferred phase for the display of activity in different animal models represents an important area to explore with respect to the cost of activity during the night, both in night work and in human eveningness.

## Ethics Statement

The work from our group discussed here was carried out in accordance with the recommendations of the Michigan State University All University Committee on Animal Use and Care, and the National Institute of Health guide for the Care and Use of Laboratory Animals. All protocols were approved by the Michigan State University All University Committee on Animal Use and Care.

## Author Contributions

The ideas presented here stem from discussions among the three authors, AN wrote the first draft and LS and LY edited and expanded the initial version.

## Conflict of Interest Statement

The authors declare that the research was conducted in the absence of any commercial or financial relationships that could be construed as a potential conflict of interest.
